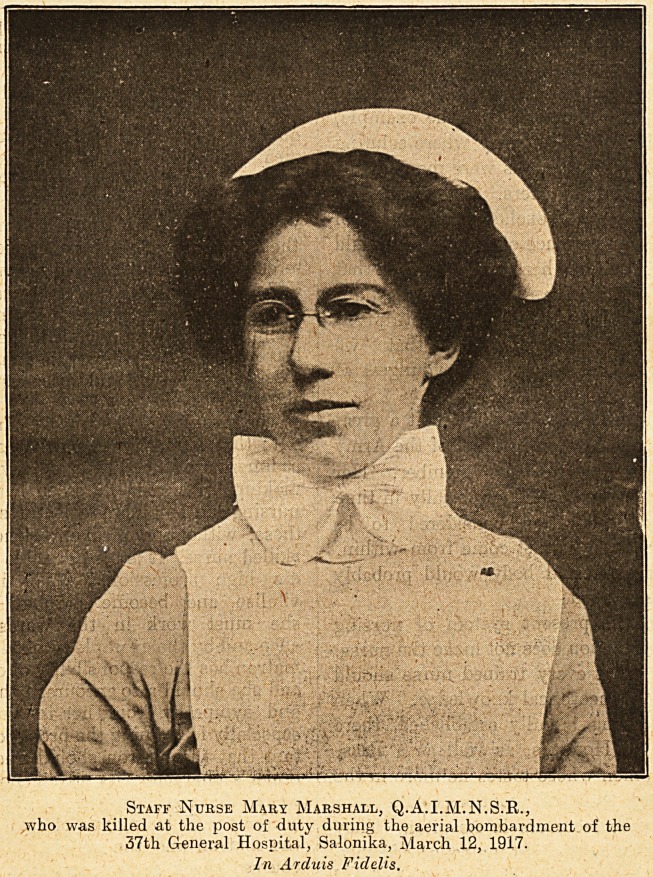# Round the Hospitals

**Published:** 1917-08-11

**Authors:** 


### ROUND THE HOSPITALS.

HER MAJESTY QUEEN MARY is so full of energy, and has worked so hard, especially during the three trying years of the worst war, that her absence from the Service of Intercession at the Abbey on Sunday last came almost as a shock to His Majesty's loyal subjects. We are glad to know that the indisposition, though temporary in character, has won for Her Majesty the sympathy of all workers amongst the sick, who are able from experience to estimate the strain of the war upon the individual worker and to realise that prudence demands exceptional care to avoid risks, if the maximum of health is to be continuously won.

ON page 375 of this issue matrons and sisters will find an interesting article on "The Worst War in the World: Its Third Anniversary in Westminster Abbey," and on p. 376 an account of the Abbey Service on the 5th inst., with some interesting facts about the service, music, and hymns. Particulars of the new War Hymn, "O Valiant Hearts," by John S. Arkwright, will also be found on p. 376, and the words of the hymn on p. iv. Everyone who takes a living interest in the Hospital Chapel and its services, and values both, may find food for thought and use in the articles we refer to.

WHEN the German pirates commenced deliberately to sink hospital ships, and aggravated their brutal criminality by lying excuses, British-trained nurses felt, and we agreed with them, that if hospital ships were kept in continuous use they should in the ordinary course of duty face the risk and attend to the wounded on board. The authorities decided not to carry trained nurses on hospital ships, however, to the grief of the best of our British nurses and of those who know most about their courageous spirit and their standards of work. As we go to press it is announced from Madrid that, as a result of representations made by the Spanish Government in Berlin, London, and Paris, the German Government has agreed to a safe passage for hospital ships, on condition that a Spanish naval officer is on board who guarantees that the vessel is being used only for the transport of the sick and wounded. The *Heraldo*, a Spanish newspaper, adds that the French and British Governments have agreed to this arrangement, and that eleven Spanish naval officers are to be sent to various ports which shall be indicated by the Allies. If this report is confirmed British-trained nurses connected with the transport department of the Services and the Red Cross organisation will be able to take up their work on behalf of the wounded, to the advantage of our brave warriors.

STAFF NURSE MARY MARSHALL, Q.A.I.M.N.S.R., who was killed at the post of duty during the aerial bombardment of the 37th General Hospital, Salonika, March 12, 1917.
*In Arduis Fidelis.*

WE have received a copy of the report of the Nightingale Fund for 1916. The great fact of the report is a statement by the matron that the final examination of nurses who have gone through the whole three years' course took place in July 1916, with satisfactory results. No award of the Gold Medal was made, but representatives of the examiners, Dr. Turney and Dr. Russell, allotted the marks, with the result that the remaining Nightingale Medals were awarded as follows: The Silver Medal to Miss Dorothy Bannon, and the Bronze to

Miss Persis Stagg. Miss Hannah Brooks obtained honourable mention. The matron expresses the gratitude of the nursing staff to the founder of these medals, Mrs. Minet, herself trained in the Nightingale School. The Nightingale School will ever be grateful for her gift, which will open a wide future of work and interest to the holders, apart from the impetus it has given both to pupils and teachers during these courses of study. On the latter point we hope to have the privilege of testifying in an early issue, when we shall take an opportunity of dealing fully with the Nightingale Fund Report for 1916.

THE zeal of Miss Rundle for her work and the interests of the College of Nursing, Limited, were demonstrated by her attendance at a meeting at the Hull Royal Infirmary on July 31, on her way to the holiday resort of her choice in Scotland. The chair was taken by Mr. Arthur J. Atkinson, Sheriff of Hull, who is chairman of the House Committee of the Royal Infirmary, and the nursing institutions of the city of Hull were well represented by the matrons and members of their staffs. Miss Rundle explained the objects of the College in a very animated and interesting manner, and so moved a member of the Children's Hospital Committee, Councillor Sheardown, that a lively discussion ensued on the question, Why is a nurse trained in a children's hospital not eligible for membership and enrolment on the College Register? The reporters on whom we rely for this notice show commendable reserve by not intimating whether the lady or the man came off triumphant in the debate just mentioned. Votes of thanks were passed to the Chairman, and the Misses Harrison, Prichard, and Little, in addition to one to Miss Rundle for her attendance and address.

THE authorities of the Royal Devon and Exeter Hospital, Exeter, have decided to present those sisters and nurses who have been trained at this institution with a badge of round design, mounted in enamel on bronze and suitably inscribed, as a souvenir to mark their period of training at Exeter. The principle will not be made retrospective, but former nurses of the hospital, desirous of possessing one of these badges, are entitled to purchase it at cost price on their making application to the Secretary, Mr. Samuel S. Cole, to whom we are indebted for this information.

IT is a generally accepted view that there is nothing in a name. Dean Wace, of Canterbury, at a recent meeting, maintained the contrary. "It was first suggested to call the Linen League of the local hospital the Ladies' Linen League, but ultimately the shorter title was approved, because men joined them in the work; so the Canterbury Linen League had set an example which had recently been followed in the House of Commons."

IT is a notable fact, worthy of being recorded and remembered, that the London Munitions Tribunal has laid down the rule that a nurse engaged in nursing duties at a controlled establishment does not require a leaving certificate in order to vacate her position. On the other hand, in contrast to this, we may mention that the County Court Judge at Marylebone, Sir William Selfe, on the 25th ult., ruled that a nurse who goes out to cases on her own account cannot claim under the Workmen's Compensation Act, for the reasons (1) that she did not contract for service, but for services rendered; (2) that her employment was of a casual nature, inasmuch as a nurse was called in only as occasion arose, and was not a part of the regular staff of an establishment. Trained nurses will, therefore, be well advised to insure against accidents when so engaged.

### Matrons in Council.

### SPARKS FROM A SISTER'S ANVIL.

"WONDERING" sends us the following letter, which we hope is one of a great many more to follow from matrons as well as sisters :—

"As a sister I am most interested in last week's article, 'Sparks from a Nurse's Anvil.' For some time past I have been wondering if there is a great tendency on the part of matrons and sisters to be not exactly slack, but shall we say more lenient, and to overlook little things which in former days it would have been a serious offence to commit.

"This attitude, I think, is partly due to the fact that there is a great deal of extra work to be done, especially in those hospitals where accommodation has been made for soldiers, and where a depleted staff has been trying to cope with extra patients with what extra help could be obtained from military nurses.

"Some of these nurses are only partly trained, and as military nurses they are regarded in a somewhat different light to the probationers who are now being trained, and of whom we expect so much in the future.

"Little points of etiquette are overlooked, rules become more or less elastic. In these strenuous times we are all apt to get a little 'weary in well doing,' and unless we keep a good grip of our ordinary work by 'ordinary work'—I mean the discipline and efficient training of probationers as carried out before the war—all the sins of omission that come into existence now will lower our standard of nursing instead of raising it."

### The "Choker" Collar: Another View.

I. M. writes : "I agree with a Red Cross matron that the ordinary starched high collars generally worn by nurses are most uncomfortable, and my experience is that most nurses dislike them very much, and hail with delight any change which is at the same time smart and comfortable. When I was matron of a large hospital I had collars specially made with a curve over the shoulder, and shorter at the front than at the back; and later, during one very hot summer, our own sewing-maids made 'Peter Pan' collars out of fine drill. These looked quite smart, and were so much liked that we never reverted to the starched collars. In my present hospital my nurses wear ordinary Eton collars, which look very nice and do not restrict the neck in any way. During the recent hot weather several of them have told me how much pleasanter and easier it was to work than in the 'high choker' days."

## Figures and Tables

**Figure f1:**